# 非小细胞肺癌中驱动基因状态与免疫治疗相关性的研究进展

**DOI:** 10.3779/j.issn.1009-3419.2019.04.06

**Published:** 2019-04-20

**Authors:** 捷 陈, 达 姜, 芳 黄

**Affiliations:** 050011 石家庄，河北医科大学第四医院肿瘤内科 The Forth Hospital of Hebei Medical University, Shijiazhuang 050011, China

**Keywords:** 肺肿瘤, 免疫治疗, 驱动基因, 超进展, Lung neoplasms, Immnotherapy, Driver Gene, Hyperprogression

## Abstract

近几年，针对阻断程序性死亡蛋白1（programmed cell death 1, PD-1）及其配体1（PD-1 ligand, PD-L1）的免疫检查点抑制剂在非小细胞肺癌（non-small cell lung cancer, NSCLC）治疗中取得了里程碑式的意义。然而目前的免疫治疗还不够精准，在未经选择人群中有效率偏低，只有15%-20%患者能从中获益，且有发生超进展（hyperprogression, HP）的几率。因此，如何选择优势人群至关重要。虽然很多研究强调了肿瘤细胞表面PD-L1表达水平及肿瘤突变负荷（tumor mutation burden, TMB）检测等指标指导免疫治疗的重要性，然而基于各种障碍，目前PD-L1表达水平及TMB尚不能作为决定性、排除性的预测标志物。随着研究深入，我们发现肺癌驱动基因突变与PD-1/PD-L1信号的异常激活之间有着密切的联系，基因突变与免疫治疗疗效的相关性尚有广阔研究价值。本文就基因突变预测免疫检查点抑制剂疗效及HP可能的研究进展作一综述。

## Abstract (en)

In recent years, the checkpoint inhibitors targeted programmed cell death 1 (PD-1) and its ligand 1 (PD-1 ligand, PD-L1) achieved landmark significance in treating a variety of cancers including non-small cell lung cancer (NSCLC). However, current immunotherapy is not precise enough, only 15%-20% of the unselected patients can benefit from the therapy, and there is a possibility of hyperprogression (HP). Therefore, how to select the dominant population is crucial. Although many studies have emphasized the importance of PD-L1 and tumor mutation burden (TMB) and other indicators to guide immunotherapy, current PD-L1 expression level and mutation load cannot be used as a decisive and excluded predictive marker based on various obstacles. With the deepening of research, we found that there is a close relationship between lung cancer-driver gene mutation and aberrant activation of PD-1/PD-L1 signal pathways, and the correlation between gene mutation and immunotherapy efficacy has broad research value. This article will revolve around the above issues.

肺癌是中国及全球范围内发病率最高的实体肿瘤，中国患者中肺癌的5年生存率仅有19.7%^[1]^，而肺癌中约80%以上的患者为非小细胞肺癌（non-small cell lung cancer, NSCLC）。含铂双药化疗作为晚期NSCLC的标准治疗方案，其发展也进入了瓶颈期，以表皮生长因子酪氨酸激酶抑制剂（epidermal growth factor receptor-tyrosine kinase inhibitors, EGFR-TKIs）为代表的分子靶向治疗显著延长了驱动基因阳性的晚期NSCLC的无进展生存期（progression-free survival, PFS）及总生存期（overall survival, OS）^[2]^，但是驱动基因作为与NSCLC发生发展相关的重要基因，其不稳定性决定了靶向药物治疗难以治愈肿瘤。免疫治疗作为治疗肿瘤的新希望，将晚期NSCLC 5年生存率呈数倍提升，然而其在未经选择人群中也只有15%-20%患者能从中获益，且有9%-29%的患者会出现HP，预后极度不良^[3]^。所以在免疫治疗的精准之路上仍需进一步探索。随着研究深入，肿瘤驱动基因通路和PD-1/PD-L1通路可能存在相互作用，众多研究显示对于驱动基因阳性的肺癌患者，免疫治疗效果差强人意，相关权威指南也将肺癌免疫治疗的人群限制在无驱动基因突变的患者。那么，是否能明确驱动基因阳性与免疫检查点抑制剂在晚期NSCLC患者中的疗效呈负相关，驱动基因和其他疗效预测标志物的内在联系有哪些，作用机制有哪些，基因突变状态能否预测HP，本文就这些问题的研究进展作一综述。

## 1 驱动基因突变状态预测免疫治疗疗效

### 1.1 EGFR

*EGFR*是NSCLC中最重要的驱动基因之一。据统计47.9%的亚洲NSCLC患者携带*EGFR*突变，并且EGFR检测方法成熟而规范，其通路与PD-1/PD-L1通路的关系密不可分。众多研究表明*EGFR*突变患者免疫治疗获益不明显。Borghaei等^[4]^的研究是Nivolumab对比多西他赛用于二线的非鳞状NSCLC的疗效分析，其主要研究终点OS的亚组分析显示，EGFR野生型患者较突变型患者从Nivolumab治疗中获得较好生存，然而*EGFR*突变组的HR（95%CI）为1.18（0.69-2.00），因此这一结论受限于小样本突变组HR的过于广泛的可信区间以及突变组数据的不完全统计，因此不能说明EGFR野生型患者从免疫治疗中获益。Herbst等^[5]^研究了Pembrolizumab对比多西他赛用于经治的、PD-L1阳性细胞表达率≥1%的晚期NSCLC。其中有9%的*EGFR*突变患者，这些患者并没有从免疫治疗中得到明显的临床获益。在一些*meta*分析的亚组分析中也涉及到了在不同*EGFR*突变状态下免疫检查点抑制剂对比化疗在总生存期及中位生存期上的差异。一项研究^[6]^中入组了4项Ⅲ期随机临床实验（使用的免疫检查点抑制剂包括atezolizumab、nivolumab、pembrolizumab），亚组分析显示，*EGFR*野生组人群比突变组人群更可能有OS获益（*P*=0.05）。其中*EGFR*突变人群占总人群的11.2%，此实验不能除外因小样本引起的Ⅱ型错误，并且一些实验没有给出*EGFR*突变状态，所以有可能会影响结果的可靠性。另一项*meta*分析^[7]^共入组9个相关实验，发现不仅PD-L1表达水平，还有*EGFR*突变状态也可影响免疫检查点抑制剂的疗效。而Lee等^[8]^的研究入组了3项随机临床试验，显示在*EGFR*突变组免疫检查点抑制剂对比多西他赛二线治疗晚期NSCLC无OS获益（*n*=186, HR: 1.05, *P* < 0.81）。综合上述研究结果，*EGFR*突变组NSCLC患者对比野生组患者对于免疫检查点抑制剂治疗的反应率低。然而，这些实验有局限性，其中有小样本实验，有回顾性研究，*meta*分析中各实验存在异质性，入组标准存在差异，且各实验*EGFR*突变组的样本量远远小于野生组，其结论尚需进一步数据理论支持。

无法忽略的一点是，*EGFR*突变组缺乏免疫治疗获益也可能是由于低TMB或者是低PD-L1表达，或是导致了免疫抑制性微环境，那么，经多线治疗后，随着肿瘤细胞免疫原性的增加，是否有可能使得免疫检查点抑制剂获得更好的疗效呢？ATLANTIC研究^[9]^是一项2期单臂临床实验，研究结果显示，*EGFR*/*ALK*突变患者，如果PD-L1阳性细胞表达率≥25%，应用Durvalumab治疗总的生存获益达到13.3个月，比EGFR/ALK野生型且PD-L1阳性细胞表达率≥25%的亚组还多2.4个月。因此，该项研究认为，多线研究后再次使用Durvalumab，无论EGFR/ALK状态如何，均能从治疗中取得生存获益。因此，*EGFR*突变伴PD-L1高表达人群可能仍适于免疫治疗。

#### 1.1.1 *EGFR*突变与PD-L1表达的关系

有一项II期研究结果显示，*EGFR*突变中PD-L1高表达的患者相对较少，大约只有5%^[10]^。此外在CheckMate 012研究^[11]^中，8例*EGFR*突变的NSCLC患者，4例对免疫治疗产生应答，其中1例患者PD-L1阳性细胞表达率≥1%，3例PD-L1阳性细胞表达率≥50%。这些结果均提示PD-L1表达水平低的*EGFR*突变患者免疫治疗疗效较差，结合上述的ATLANTIC研究，*EGFR*突变伴PD-L1高表达人群可能仍适于免疫治疗。

而关于*EGFR*突变对PD-L1表达水平的影响众说纷纭。很多研究结果显示PD-L1的表达受到EGFR信号的调控，然而*EGFR*突变是上调还是下调PD-L1表达仍有争议。Chen等^[12]^的研究未观察到EGFR-TKIs和抗PD-1抗体联合治疗的协同肿瘤细胞杀伤效果，但认为EGFR可上调PD-L1的表达水平。EGFR能通过p-ERK1/2/p-c-Jun通路来调控PD-L1的表达，*EGFR*基因突变会使得EGFR磷酸化水平提高，激活EGFR通路，磷酸化ERK及c-Jun，从而上调PD-L1的表达水平，而AKT通路在这个过程中并不发挥作用。Azuma等^[13]^分析了164个NSCLC术后标本的免疫组化，多样性分析显示*EGFR*突变状态与PD-L1高表达显著相关, 认为*EGFR*突变是上调*PD-L1*基因及蛋白表达的独立因素。在其他一些小样本研究^[14-16]^也发现*EGFR*突变型NSCLC患者细胞表面的PD-L1表达水平比野生型要高。因此*EGFR*突变阳性可能会通过上调PD-L1表达发生免疫逃逸。但也有研究^[17]^表明，PI3K-AKT通路与MEK-ERK通路均参与了EGFR对PD-L1的调控，利用PI3K-AKT通路及MEK-ERK通路的特异性阻断剂，均能够观察到PD-L1表达水平的显著下调。Hata等^[18]^分析了PD-L1表达与EGFR突变状态的关联。研究入组了77例经治NSCLC患者的96个标本，结果显示PD-L1的表达在EGFR突变的NSCLC样本中明显低于EGFR野生型。该研究认为，PD-L1表达与*EGFR*突变的关系仍有争议可能与EGFR-TKIs治疗手段介入导致的异质性相关。此外一些小样本研究^[19-20]^也认为*EGFR*突变患者的*PD-L1*基因及蛋白表达水平下降。

然而也有很多研究表明，PD-L1的表达与EGFR通路并无相关性，*EGFR*突变没有增加或减少PD-L1的表达。Yang等^[21]^检测了143例手术切除的肺癌组织标本，在*EGFR*突变型肺癌组织中发现PD-L1和PD-L2表达阳性与阴性的例数相比并无显著性差距。一些其他研究^[29]^也得出了相似的结论。为什么不同的研究得出如此迥异的研究结果呢？原因可能与PD-L1抗体检测的多样性及cutoff值不一致有关，另一个原因可能是不同人种及分子谱的差异，在欧美人群与东亚人群中，*EGFR*突变率存在差异。因此还需要更加深入的研究，以明确EGFR与PD-L1表达之间的关系及具体机制。

#### 1.1.2 *EGFR*突变与TMB的关系

*EGFR*突变组免疫治疗效果差，一些学者认为最可能的解释是*EGFR*突变产生了弱的免疫原性和能导致免疫抑制的肿瘤微环境或者说是非炎性的肿瘤微环境。有研究^[6]^显示了*EGFR*突变组NSCLC患者对比EGFR野生组患者有较低的突变负荷，这一更低的突变负荷可以导致对免疫检查点抑制剂更低的敏感性。这一联系背后的机制可能是，可以被效应T细胞识别的体细胞突变导致了细胞质新生抗原的过度表达。*EGFR*突变尽管存在驱动基因阳性，但是产生新抗原的能力差。而Dong等^[19]^的研究结果体现了这一预测的可能性。驱动基因突变很少产生新生抗原，在20, 000个黑色素瘤内发现了20种新生抗原，只有8%的新生抗原来自驱动基因的突变，而剩下的92%的新生抗原均来自非驱动基因突变。此外，不同突变类型产生新抗原的能力不一致，对新生抗原的免疫原性也有影响。上述研究发现，*EGFR*突变比其他突变有最低的突变负荷和最低的Tv/Ti比率。因此，*EGFR*突变患者接受免疫治疗获益较差可能与此相关。

除了与免疫抑制分子相关外，研究认为具有*EGFR*突变的肿瘤多表现为单一致癌信号通路的活化，这类肿瘤较少产生其它基因的变异。另外，也有研究^[19]^显示，*EGFR*突变是吸烟人群中TMB减低的主要因素。因此*EGFR*突变肿瘤被认为是一种低免疫原性肿瘤，并不适合免疫治疗。也有研究认为*EGFR*突变患者有较低的TIL，导致机体由于缺乏效应细胞限制了免疫治疗的疗效。结合目前的研究数据，*EGFR*突变型NSCLC人群可能不是接受免疫检查点抑制剂治疗的最佳人群，然而免疫治疗绝不是*EGFR*突变患者的禁地，如*EGFR*突变伴PD-L1高表达人群可能仍适于免疫检查点抑制剂治疗。

### 1.2 间变性淋巴瘤激酶（anaplastic lymphoma kinase, ALK）

*ALK*也是NSCLC重要的驱动基因，其中最常见的是棘皮动物微管相关蛋白4（EML4）-*ALK*融合基因突变，中国NSCLC患者中的阳性率为3%-7%。ALK与EGFR类似，均可通过激活PI3K/AKT及MEK/ERK信号通路上调PD-L1表达。有研究^[13]^发现，EML4-ALK阳性细胞的PD-L1表达水平显著高于EML4-ALK阴性的细胞，并且EML4-ALK融合蛋白的表达能够上调PD-L1的表达，抑制ALK的活性或敲除EML4-ALK后，同样能够降低PD-L1的表达。这表明PD-L1的表达受到EML4-ALK融合蛋白的调控。另有数据^[22]^表明，*EGFR*突变和*ALK*重排患者有较短的PFS和低ORR，表明*ALK*重排患者免疫治疗类似于*EGFR*突变患者，同样没有显示出确切的疗效，而PD-L1和CD8^+^ TILs共同存在的低比率可能是这一结论的基础。上文提到的ATLANTIC研究^[9]^显示，对于ALK重排患者，如果PD-L1阳性细胞表达率≥25%，那么免疫治疗也会起效。但EGFR与ALK免疫生物学性质可能有所不同。这足以说明*ALK*基因通路与免疫微环境之间相互作用的复杂性，免疫治疗在*ALK*重排的NSCLC患者中疗效并不突出。

### 1.3 KRAS/TP53

*KRAS*基因是NSCLC中重要的驱动基因之一，在NSCLC患者中，有15%-20%携带有*KRAS*基因突变，而且常发生在吸烟的肺腺癌患者中。*KRAS*基因属于*RAS*基因家族，为EGFR信号下游重要的调节位点，参与细胞增殖与分化的调节。*KRAS*基因突变使得在缺乏生长因子信号的条件下也可激活RAS-GTP酶，参与肿瘤的生成、增值、迁移、扩散以及血管生成各个方面，并且致癌RAS信号能通过稳定PD-L1 mRNA使得机体产生免疫耐受，*RAS*基因突变可以通过下游信号维持编码PD-L1蛋白的mRNA稳定，癌细胞不断合成PD-L1，与T细胞表面的PD-1结合，从而使癌细胞具备免疫抑制能力，发生免疫逃逸据。D’Incecco等^[23]^的研究观察到，PD-L1的表达与*KRAS*基因突变存在显著的相关性（*P*=0.006）。因此，*KRAS*基因与免疫微环境之间有何关联？*KRAS*基因突变患者与野生型患者对于免疫治疗的生存期有无差异？Dong等^[19]^研究了*KRAS*基因突变状态对肺腺癌中PD-1阻断治疗的研究价值。研究发现，*KRAS*突变患者PD-L1表达下降，并且具有更高的TMB、更高的Tv/Ti比率和错配修复基因缺失。一项回顾性研究^[24]^也发现，高*KRAS*突变率的患者低表达PD-L1。这些研究结果基本一致，指出了*KRAS*突变患者可能低表达PD-L1并且具有高的体细胞突变负荷。但是也有研究认为KRAS被认为与PD-L1表达呈正相关，而KRAS信号可能不参与PD-L1表达的调控^[25]^。因此这其中的复杂关系仍有争议，尚需要样本量更大、更权威的实验去进一步证实。但上述研究也提示*KRAS*突变患者可能获益于抗PD-1/PD-L1治疗。

从CheckMate 057实验^[4]^中可以看出，携带*KRAS*基因突变的患者，接受Nivolumab治疗之后获益，那些没有*KRAS*突变的患者没有从治疗中获益。Dong等^[19]^也指出，在免疫治疗中，*KRAS*突变与免疫治疗疗效的相关性非常复杂。*KRAS*突变有3种类型：仅*KRAS*突变、*KRAS*/*TP53*共突变、*KRAS*/*STK11*共突变。研究回顾性分析了接受过免疫治疗且存在*KRAS*突变的174例肺腺癌患者，得出结论：如果单纯*KRAS*突变，可能会预测此部分患者对于免疫治疗有更好的疗效，*KRAS*/*TP53*共突变患者能最大程度临床获益（ORR为30%），即使PD-L1表达为阳性也不例外。而*KRAS*/*STK11*共突变患者获益最少，提示*STK11*基因异常可能是*KRAS*突变的肺腺癌患者对免疫检查点抑制剂原发耐药的主要原因。很多人认为，之所以得出*KRAS*突变NSCLC患者免疫治疗疗效较好的结论，并且突变患者的TMB较高，均可能和吸烟相关。众所周知，吸烟是导致肿瘤发生突变的一个重要因素，吸烟相关肺癌被认为与TMB及*KRAS*突变发生率密切相关，吸烟患者中*TP53*突变是导致PD-L1表达上调的独立因素，STK11是下调因素，而非吸烟患者中任何基因突变均未改变PD-L1表达。这提示吸烟患者发生发生*TP53*突变是PD-L1表达上调的主要人群，而KRAS不是其显著的相关因素，并且*KRAS*突变仅在不吸烟人群中导致TMB增加。

由此可见，*KRAS*基因与PD-1通路之间具有复杂的相关性，多数学者认为在NSCLC患者中，*KRAS*突变作为独立因素有更低的PD-L1表达及更高的突变负荷，且*KRAS*突变比野生型可能免疫治疗效果好，*KRAS*突变状态有望成为NSCLC中免疫治疗疗效的预测标志物，但目前缺乏进一步大样本前瞻性验证。

### 1.4 MUC16

来自天津市肿瘤研究所的研究人员发现^[26]^，在437例美国胃癌患者与256例亚洲胃癌患者的基因检测样本中，在168例（38%）美国样品组和57例（22%）亚洲样本组观察到*MUC16*基因突变。由于*MUC16*基因能够编码肿瘤抗原，此基因的突变会产生新的肿瘤抗原，因此MUC16患者有更高的TMB水平。*MUC16*基因在包括胃癌及NSCLC的多种肿瘤患者中也常发生突变，基于基因检测对比TMB的可行性及方便性，*MUC16*基因作为预测免疫治疗疗效的标志物值得进一步探索。

### 1.5 其他基因

大约有2%-4%NSCLC患者携带*MET*外显子14突变，此类患者通常有吸烟史，且同时PD-L1表达高以及有更多的肿瘤免疫细胞浸润，但这部分患者TMB不高，对免疫治疗的反应并不高，目前这方面研究仍处于初级阶段，其潜在的相关机制尚不明朗。研究^[27]^显示，BRCA1/2及*POLE*突变的NSCLC患者有更高的TMB。Rizvi等^[28]^也在TMB最高的NSCLC患者中发现了*POLD1*、*POLE*以及*MSH2*突变。此外，*STK11*基因缺失，肿瘤组织中很少见到有效应性T淋巴细胞的浸润，也就是冷肿瘤，因此免疫治疗疗效欠佳，STK11缺失人群PD-L1表达通常较低，即使是TMB高负荷，也无法从免疫治疗中获益。TP53可能增加TMB，提高PD-L1表达，*TP53*突变肿瘤可能有更好的免疫疗效^[29]^。这些罕见基因的突变状态与免疫治疗的相关性也在研究进行中，期待取得更多的突破。

## 2 驱动基因突变状态预测免疫治疗超进展

免疫检查点抑制剂治疗成果鼓舞人心，但仍有部分患者在接受免疫治疗期间出现超进展（hyperprogression, HP）现象。HP目前尚无标准定义，综合目前文献，HP被定义为肿瘤的反常加速生长。包括在免疫治疗后第一次评价时进展，肿瘤体积增加 > 50%且肿瘤增长速度 > 2倍。接受免疫检查点抑制剂治疗后疾病HP的发生率为9%-29%，这部分患者一般预后极差，中位生存时间仅仅有2个月-5个月^[3, 30]^。因此，如何预测HP的发生至关重要。HP的分子机制仍然非常难以捉摸，在基因组生物标志物方面缺乏数据。只有*MDM2*/*MDM4*（murine double minute homolog 2 and 4）扩增和*EGFR*突变已被证实为HP独立的相关因素。就*MDM2*/*MDM4*扩增而言，Kato和Singavi等研究^[31]^发现，大约2/3的*MDM2*/*MDM4*扩增患者在队列中发现了HP现象，肿瘤进展速度高达40倍。然而还不能排除*MDM2*扩增子上不同基因的共扩增是HP的真正预测标志物。对于*EGFR*基因突变，在上述研究^[31]^中，有20%（2/10）和50%（1/2）的*EGFR*突变患者被证实为免疫治疗HP患者，其肿瘤进展速度亦可增加40倍。使免疫治疗生存获益最大化、适用人群精准化，需尽可能筛选出免疫治疗HP人群，进一步探索相关基因与HP现象的关联。

## 3 总结与展望

以PD-1/PD-L1为代表的免疫治疗作为最有希望治愈肿瘤的治疗方法，其相关研究方兴未艾。其中，有效率较低是目前限制患者获益的主要阻碍。基于目前研究结果，驱动基因阳性的NSCLC患者较阴性患者免疫治疗获益小，此部分患者目前首选的临床治疗策略是以TKI为代表的靶向治疗，驱动基因的靶标检测已相对成熟，且取材简单、可动态监测，结合NSCLC中基因突变通路与PD-1通路的相关性，驱动基因突变状态在免疫治疗中的疗效预测价值有着无限的可能性。免疫疗效和特定基因、抑癌基因及总体突变相关基因通路的关系的研究较少，且大部分为小样本或者回顾性研究，目前没有确切证据证明有特定的致癌基因或者抑癌基因作为独立变量与免疫治疗疗效相关。尚需进一步数据支持驱动基因突变作为预测标志物的地位，并进一步研究突变如何影响免疫微环境。对于驱动基因阳性的NSCLC患者，未来以免疫治疗为基础的联合治疗是一个值得展望的新方向，基于肿瘤微环境中的免疫细胞与血管生成相互作用，免疫联合治疗（如免疫检查点抑制剂联合化疗），或联合血管靶向治疗可能提高驱动基因阳性患者获益，但需注意安全性问题，警惕毒性的叠加。总之，精准治疗是今后肺癌治疗的一个方向，精准地筛选出抗PD-1/PD-L1治疗的最大获益人群还任重道远，未来仍需要一系列前瞻性、大样本、设计严格的临床研究来探索抗PD-1/PD-L1免疫治疗疗效的预测标志物。相信在精准指标的指导下，晚期NSCLC患者会得到更高效、更安全的PD-1/PD-L1抑制剂的治疗。

## References

[1] Zeng H, Chen W, Zheng R (2018). changing cancer survival in China during 2003-15: a pooled analysis of 17 population-based cancer registries. Lancet Global Health.

[2] Zhou C, Wu YL, Chen G (2011). Erlotinib versus chemotherapy as first-line treatment for patients with advanced EGFR mutation-positive non-small cell lung cancer (OPTIMAL, CTONG-0802): a multicentre, openlabel, randomised, phase 3 study. Lancet Oncol.

[3] Champiat S, Dercle L, Ammari S (2017). Hyperprogressive disease is a new pattern of progression in cancer patients treated by anti-PD-1/PD-L1. Clin Cancer Res.

[4] Borghaei H, Paz-Ares L, Horn L (2015). Nivolumab versus docetaxel in advanced nonsquamous non-small-cell lung cancer. N Engl J Med.

[5] Martin R, Rodriguez-Abreu D, Robinson AG (2016). Pembrolizumab versus docetaxel for previously treated, PD-L1-positive, advanced non-small-cell lung cancer (KEYNOTE-010): A randomised controlled trial. Lancet.

[6] Ramos-Esquivel A, Van d L A, Rojas-Vigott R (2017). Anti-PD-1/anti-PD-L1 immunotherapy versus docetaxel for previously treated advanced non-small cell lung cancer: a systematic review and meta-analysis of randomised clinical trials. ESMO Open.

[7] Wang C, Yu X, Wang W (2016). A meta-analysis of efficacy and safety of antibodies targeting PD-1/PD-L1 in treatment of advanced nonsmall cell lung cancer. Medicine.

[8] Lee C K, Man J, Lord S (2017). Checkpoint inhibitors in metastatic EGFR-mutated non-small cell lung cancer: A meta-analysis. J Thorac Oncol.

[9] Garassino M C, Cho B C, Kim J H (2018). Durvalumab as third-line or later treatment for advanced non-small-cell lung cancer (ATLANTIC): an open-label, single-arm, phase 2 study. Lancet Oncol.

[10] Peters S, Gettinger S, Johnson ML (2017). Phase Ⅱ trial of atezolizumab as first-line or subsequent therapy for patients with programmed death-ligand 1-selected advanced non-small-cell lung cancer (BIRCH). J Clin Oncol.

[11] Hellmann MD, Rizvi NA, Goldman JW (2017). Nivolumab plus ipilimumab as first-line treatment for advanced non-small-cell lung cancer (CheckMate 012): results of an open-label, phase 1, multicohort study. Lancet Oncol.

[12] Chen N, Fang W, Zhan J (2015). Upregulation of PD-L1 by EGFR activation mediates the immune escape in EGFR-driven NSCLC: implication for optional immune targeted therapy for NSCLC patients with EGFR mutation. J Thorac Oncol.

[13] Azuma K, Ota K, Kawahara A (2014). Association of PD-L1 overexpression with activating EGFR mutations in surgically resected non-small-cell lung cancer. Ann Oncol.

[14] D'Incecco A, Andreozzi M, Ludovini V (2015). PD-1 and PD-L1 expression in molecularly selected non-small-cell lung cancer patients. Br J Cancer.

[15] Ji M, Liu Y, Li Q (2016). PD-1/PD-L1 expression in non-small-cell lung cancer and its correlation with EGFR/KRAS mutations. Cancer Biol Ther.

[16] Yanna T, Wenfeng F, Yaxiong Z (2015). The association between PD-L1 and EGFR status and the prognostic value of PD-L1 in advanced non-small cell lung cancer patients treated with EGFR-TKIs. Oncotarget.

[17] Ota K, Azuma K, Kawahara A (2015). Induction of PD-L1 expression by the EML4-ALK oncoprotein and downstream signaling pathways in non-small cell lung cancer. Clin Cancer Res.

[18] Hata A, Katakami N, Nanjo S (2017). Programmed death-ligand 1 expression according to epidermal growth factor receptor mutation status in pretreated non-small cell lung cancer. Oncotarget.

[19] Dong ZY, Zhong WZ, Zhang XC (2017). Potential predictive value of TP53 and KRAS mutation status for response to PD-1 blockade immunotherapy in lung adenocarcinoma. Clin Cancer Res.

[20] Takada K, Okamoto T, Shoji F (2016). Clinical significance of PD-L1 protein expression in surgically resected primary lung adenocarcinoma. J Thorac Oncol.

[21] Yang Z, Lei W, Yuan L (2014). Protein expression of programmed death 1 ligand 1 and ligand 2 independently predict poor prognosis in surgically resected lung adenocarcinoma. Onco Targets Ther.

[22] Gainor JF, Shaw AT, Sequist LV (2016). EGFR mutations and ALK rearrangements are associated with low response rates to PD-1 pathway blockade in non-small cell lung cancer (NSCLC): A retrospective analysis. Clin Cancer Res.

[23] D'Incecco A, Andreozzi M, Ludovini V (2015). PD-1 and PD-L1 expression in molecularly selected non-small-cell lung cancer patients. Br J Cancer.

[24] Ji M, Liu Y, Li Q (2016). PD-1/PD-L1 expression in non-small-cell lung cancer and its correlation with EGFR/KRAS mutations. Cancer Biol Ther.

[25] Calles A, Liao X, Sholl LM (2015). Expression of PD-1 and its ligands, PD-L1 and PD-L2, in smokers and never smokers with KRAS-mutant lung cancer. J Thorac Oncol.

[26] Li X, Pasche B, Zhang W (2018). Association of MUC16 mutation with tumor mutation load and outcomes in patients with gastric cancer. JAMA Oncol.

[27] Spigel DR, Schrock AB, Fabrizio D (2016). Total mutation burden (TMB) in lung cancer (LC) and relationship with response to PD-1/PD-L1 targeted therapies. J Clin Oncol.

[28] Rizvi NA, Hellmann MD, Snyder A (2015). Cancer immunology. Mutational landscape determines sensitivity to PD-1 blockade in non-small cell lung cancer. Science.

[29] Cortez MA, Ivan C, Valdecanas D (2016). PDL1 regulation by p53 via miR-34. J Natl Cancer Inst.

[30] Saâda-Bouzid E, Defaucheux C, Karabajakian A (2017). Hyperprogression during anti-PD-1/PD-L1 therapy in patients with recurrent and/or metastatic head and neck squamous cell carcinoma. Ann Oncol.

[31] Kato S, Goodman A, Walavalkar V (2017). Hyperprogressors after immunotherapy: analysis of genomic alterations associated with accelerated growth rate. Clin Cancer Res.

